# Diabetes Prevention Programme and socioeconomic inequalities in Type 2 Diabetes in England

**DOI:** 10.1093/eurpub/ckac129.159

**Published:** 2022-10-25

**Authors:** G Chatzi, W Whittaker, T Chandola, T Mason, C Soiland-Reyes, M Sutton, P Bower

**Affiliations:** 1 Social Statistics, University of Manchester, Manchester, UK; 2 Division of Population Health, University of Manchester, Manchester, UK; 3 Sociology, University of Hong Kong, Hong Kong; 4 Research and Innovation, Northern Care Alliance NHS Group, Salford, UK; 5 Centre for Primary Care and Health Services, University of Manchester, Manchester, UK

## Abstract

The National Diabetes Prevention Programme (DPP) in England is a behavioural intervention for preventing Type 2 Diabetes Mellitus (T2DM) among people with non-diabetic hyperglycaemia (NDH, HbA1c 42-47 mmol/mol or 6.0-6.4%). How this programme affects inequalities by age, gender, disability, ethnicity, or deprivation is not known. We used multinomial logistic regression models to compare population characteristics at three stages along the prevention programme pathway: prevalence of NDH [using survey data from UK Household Longitudinal Study (N = 794) and Health Survey for England (N = 1,383)]; identification in primary care and offer of the programme [using administrative data from the National Diabetes Audit (N = 1,267,350)]; and programme participation [using programme provider records (N = 98,024)]). Younger adults (aged under 40) [4% of the NDH population (95% CIs 2%-6%)] and older adults (aged 80 and above) [12% (95%CIs 10%-14%] were both underrepresented amongst DPP participants [2% of DPP participants (95%CIs 1.8%-2.2%) and 8% (95%CIs 7.7%-8.3%) respectively]. People with disabilities were underrepresented in the DPP [15% (95%CIs 14.9%-15.1%) vs 60% (95%CIs 58%-62%)] compared to the general population. People living in more deprived areas were under-represented [14% (95% CIs 13.7%-14.3%) vs 20% (95%CIs 16%-24%) in the general population]. Ethnic minorities were overrepresented [36% (95%CIs 35.8%-36.2%) vs 13% (95%CIs 9%-17%) in the general population] among DPP referrals, though the proportion dropped at programme completion stage [19% (95%CI 18.5%-19.5%)]. The DPP has the potential to reduce ethnic inequalities but may widen socioeconomic, age, and disability-related inequalities in T2DM. Whilst ethnic minority groups are overrepresented at identification and offer stage, efforts are required to support the completion of the programme. Programme providers should target underrepresented groups to ensure equitable access and narrow inequalities in T2DM.

**Key messages:**

• The DPP intervention may result in a widening of socioeconomic and disability related inequalities amongst people with NDH as the programme had fewer adults in deprived areas and with a disability.

• The programme has the potential to reduce ethnic inequalities, but efforts are required to support the completion of the programme by minority ethnic groups.

